# Detection of Inflammation-Related Melanoma Small Extracellular Vesicle (sEV) mRNA Content Using Primary Melanocyte sEVs as a Reference

**DOI:** 10.3390/ijms20051235

**Published:** 2019-03-12

**Authors:** Gina T. Bardi, Numan Al-Rayan, Jamaal L. Richie, Kavitha Yaddanapudi, Joshua L. Hood

**Affiliations:** 1 Department of Pharmacology and Toxicology, University of Louisville, Louisville, KY 40202, USA; gina.bardi@louisville.edu; 2 James Graham Brown Cancer Center, University of Louisville, Louisville, KY 40202, USA; numan@vt.edu (N.A.-R.); kavitha.yaddanapudi@louisville.edu (K.Y.); 3 Molecular Targets Program, James Graham Brown Cancer Center, University of Louisville, Louisville, KY 40202, USA; Jamaal.richie@louisville.edu; 4 Department of Medicine, University of Louisville, Louisville, KY 40202, USA; 5 Department of Microbiology and Immunology, James Graham Brown Cancer Center, University of Louisville, Louisville, KY 40202, USA

**Keywords:** melanoma, extracellular vesicle, exosome, inflammation, mRNA, biomarker

## Abstract

Melanoma-derived small extracellular vesicles (sEVs) participate in tumor pathogenesis. Tumor pathogenesis is highly dependent on inflammatory processes. Given the potential for melanoma sEVs to carry tumor biomarkers, we explored the hypothesis that they may contain inflammation-related mRNA content. Biophysical characterization showed that human primary melanocyte-derived sEVs trended toward being smaller and having less negative (more neutral) zeta potential than human melanoma sEVs (A-375, SKMEL-28, and C-32). Using primary melanocyte sEVs as the control population, RT-qPCR array results demonstrated similarities and differences in gene expression between melanoma sEV types. Upregulation of pro-angiogenic chemokine ligand CXCL1, CXCL2, and CXCL8 mRNAs in A-375 and SKMEL-28 melanoma sEVs was the most consistent finding. This paralleled increased production of CXCL1, CXCL2, and CXCL8 proteins by A-375 and SKMEL-28 sEV source cells. Overall, the use of primary melanocyte sEVs as a control sEV reference population facilitated the detection of inflammation-related melanoma sEV mRNA content.

## 1. Introduction

Melanoma relies heavily on lymphatic dissemination for growth and metastasis [1]. This process is dependent on inflammation. Inflammatory signals can be induced by melanoma production of soluble factors such as cytokines. Alternatively, or in combination, melanoma cells can produce and release extracellular vesicles (EVs).

EV nomenclature remains a work in progress and has only increased in complexity with the continual characterization of EV types and subtypes [2,3]. Given the complex heterogeneity of EV populations, they are often classified in general terms such as small EVs (sEVs), recently defined by Kowal et al. to include nanovesicles known as exosomes [4]. The density of sEVs on sucrose gradients, while not necessarily required for exosome studies, has been used as a complementary biophysical parameter to further define sEVs as exosomes [5]. Qualitative expression of CD63 on sEVs tends to support an endosomal origin for an sEV population and their likely identity as exosomes [4]. However, CD63 expression can also be absent from legitimate exosome types and subpopulations [4]. 

Shedding vesicles (ectosomes/microvesicles (MVs)) can express the same markers as exosomes and possess similar biophysical properties [3]. To address this issue, updated international society for extracellular vesicle (ISEV) guidelines recommend using “extracellular vesicle” terminology to refer to EV types co-isolated by typical EV collection methods [3]. The terms “exosome” and “ectosome/MV” are more appropriately reserved to distinguish the subcellular biogenesis of exosomes in multivesicular bodies/endosomes versus plasma membrane-generated ectosomes/MVs. For the purposes of this study, we will use the term “sEVs” to denote our melanoma EVs of interest.

Small EVs contain and transmit lipid, protein, and RNA information [6]. In recent years, pro-tumor processes attributed to melanoma sEVs include immune suppression [6], preparation of pre-metastatic niches in lymph nodes [7], education of bone marrow progenitor cells toward a pro-metastatic phenotype [8], and induction of pro-tumor macrophages [9]. Melanoma sEVs have also been found to contain and transmit angiogenic and immunomodulatory proteins such as VEGF and IL-6 to endothelial cells resulting in increased 3D endothelial tubule branching in vitro [10]. 

The ability of sEVs to contain tumor source cell information, representative of tumor function, supports the development of sEV-based biomarkers. Such biomarkers promise to facilitate earlier cancer detection, serve as prognosticators, allow for the monitoring of residual disease, or response to therapeutics. Development of melanoma sEV-based biomarkers is particularly relevant and utile given that melanoma sEVs are pathogenic, and circulatory tumor sEV burden tends to increase with tumor stage [8,11]. Given the potential for melanoma sEVs to serve as biomarkers, we explored the hypothesis that they may contain mRNA indicative of melanoma inflammatory processes. 

## 2. Results

### 2.1. Biophysical Characterization of Primary Melanocyte sEVs and Melanoma sEVs

**Figure 1:**
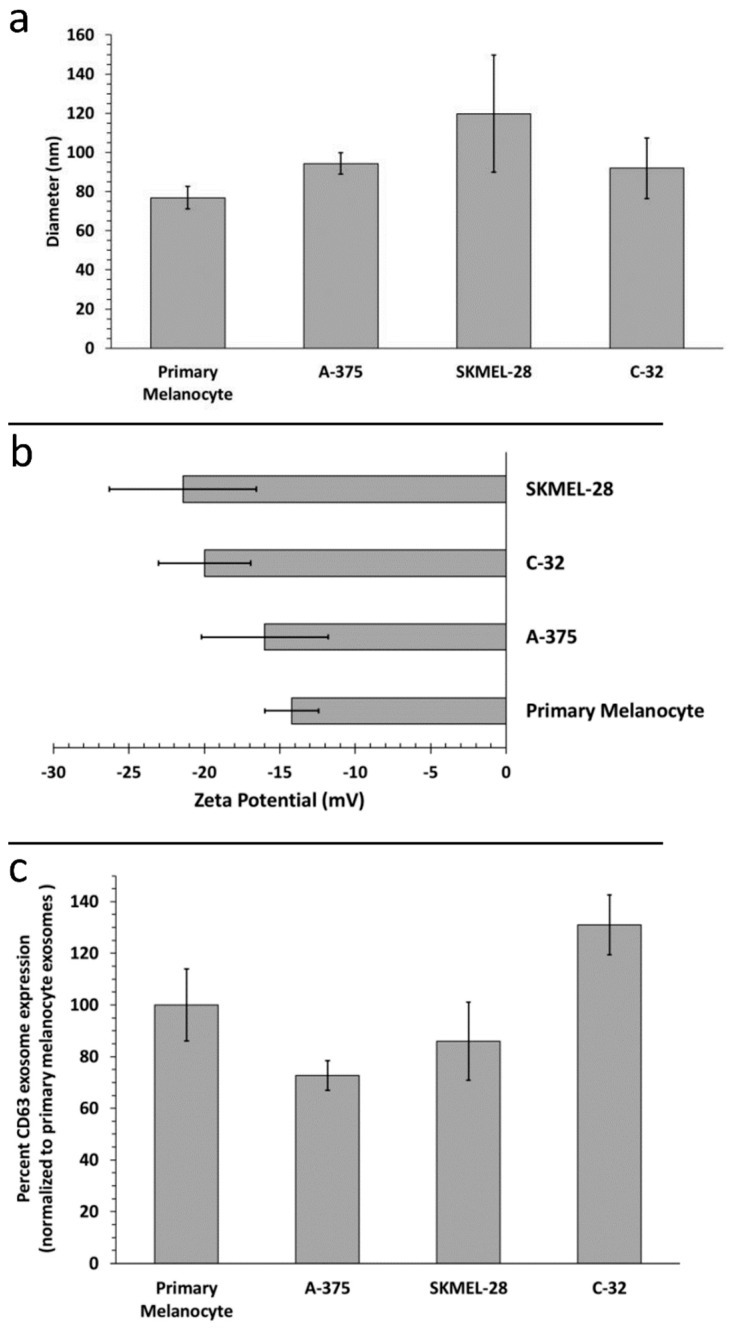
Characterization of primary melanocyte small extracellular vesicles (sEVs) and melanoma sEVs. (**a**) Small extracellular vesicle (EV) sizing by dynamic light scattering (*n =* 3 independent batch assessments), (**b**) small EV zeta potential (*n =* 3 independent batch assessments), (**c**) primary melanocyte versus melanoma sEV CD63 expression (*n =* 3 independent batch assessments). Error bars = SD, *p* values < 0.05 were considered statistically significant and were not detected.

Having isolated melanoma sEVs, we first sought to elucidate differences in melanoma sEV biophysical characteristics. We compared the biophysical properties of normal human primary melanocyte sEVs to various melanoma sEV sources: A-375 (female, age 54), SKMEL-28 (male, age 51) and C-32 (amelanotic). While melanoma sEVs trended toward being larger than primary melanocyte sEVs, no significant difference in hydrodynamic diameter between primary melanocyte sEVs and melanoma sEVs was observed (Figure 1a). All sEV size determinations were within range of what has previously been reported for sEVs [12,13]. All sEV types evaluated possessed negative zeta potentials (electrophoretic mobilities), consistent with previous reports [7,14,15,16,17] (Figure 1b). Melanoma sEVs trended toward possessing more negative zeta potentials than primary melanocyte sEVs, but the difference was not statistically significant. Further, no difference in the expression of the sEV marker CD63 was detected between primary melanocyte sEVs and melanoma sEVs (Figure 1c). 

**Figure 2:**
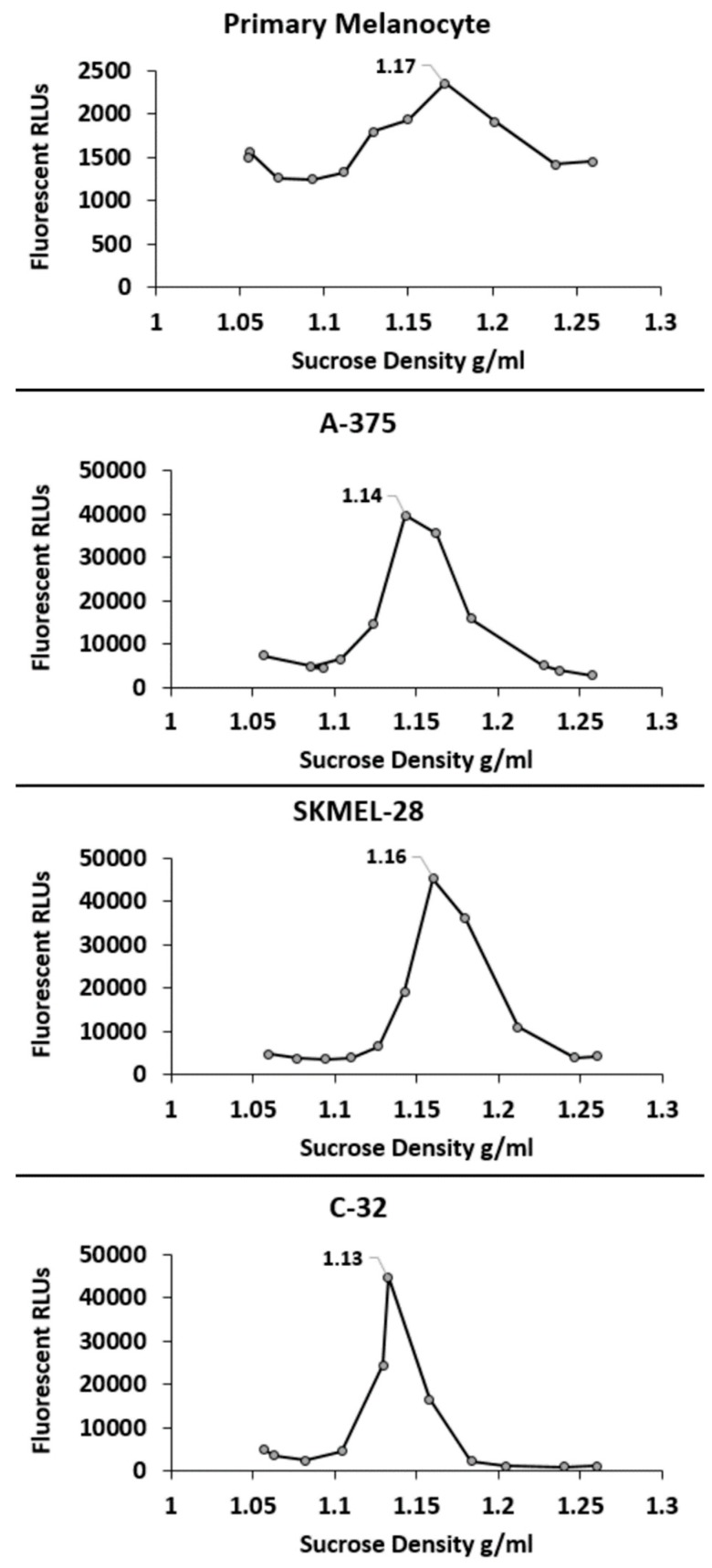
Density characterization of primary melanocyte sEVs and melanoma sEVs. Representative sucrose density gradients are shown. RLU = relative light units corresponding to sEV carbocyanine DiI signal. Peak sEV density is labeled on each gradient.

Subsequently, sucrose density gradient centrifugation of fluorescent red DiI carbocyanine labeled sEVs was employed to determine the density of the different sEV types [18]. As shown, the peak density of each of the sEV types was well within the reported range of sEV densities (~1.22–1.08 g/mL) [12] (Figure 2). 

### 2.2. Identification of Inflammation-Related mRNA Content of Primary Melanocyte sEVs and Melanoma sEVs

Following biophysical characterization of the sEVs, we proceeded to assess specific inflammation-related mRNA content using a human cancer inflammation and immunity crosstalk quantitative reverse transcription PCR array (RT-qPCR array, Qiagen). The array assesses 84 of the most well-known genes involved in mediating inflammatory signals between tumor cells and immune cells. Primary melanocyte sEVs served as the normal reference control for the melanoma sEVs. 

**Figure 3:**
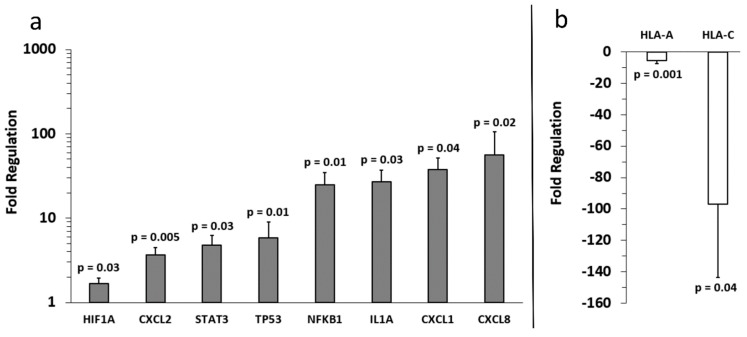
Fold regulation of A-375 melanoma sEV mRNA versus control primary melanocyte sEV mRNA. (**a**) Increased and (**b**) decreased gene expression levels relative to primary melanocytes (normalized to 1) are shown. Three independent sEV batches were pooled and run on (*n =* 3) replicate arrays, error bars = SD, *p* values < 0.05 were considered statistically significant.

**Figure 4:**
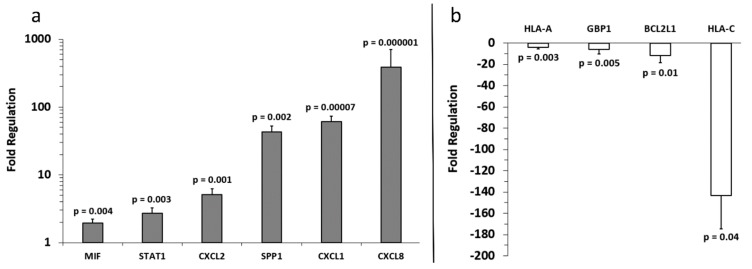
Fold regulation of SKMEL-28 melanoma sEV mRNA versus control primary melanocyte sEV mRNA. (**a**) Increased and (**b**) decreased gene expression levels relative to primary melanocytes (normalized to 1) are shown. Three independent sEV batches were pooled and run on (*n =* 3) replicate arrays, error bars = SD, *p* values < 0.05 were considered statistically significant.

**Figure 5:**
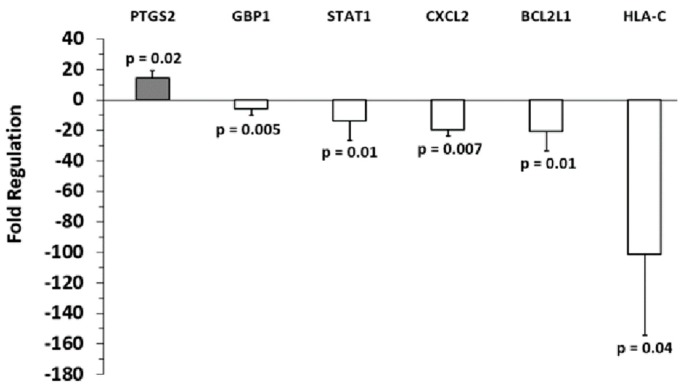
Fold regulation of C-32 melanoma sEV mRNA versus control primary melanocyte sEV mRNA. Gene expression levels for primary melanocytes (normalized to 1) are shown. Three independent sEV batches were pooled and run on (*n =* 3) replicate arrays, error bars = SD, *p* values < 0.05 were considered statistically significant.

Analysis of the array results revealed that, compared to primary melanocyte sEVs (control), more genes were positively expressed in A-375 sEVs (Figure 3) than SKMEL-28 sEVs (Figure 4). Also, more genes were positively expressed in SKMEL-28 sEVs (Figure 4) than C-32 sEVs (Figure 5). In contrast, the reverse order was observed for the negatively expressed genes. 

**Figure 6:**
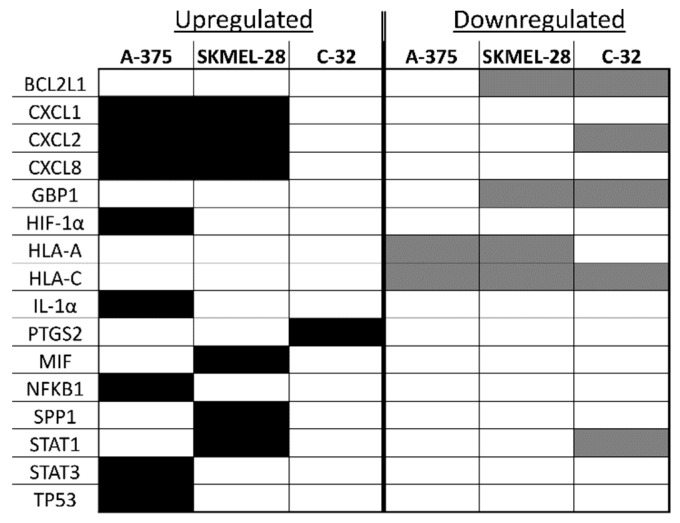
A comparison of inflammation-associated mRNA upregulated or downregulated in melanoma sEVs versus primary melanocyte sEVs. Black shaded boxes indicate upregulated gene expression, and gray shaded boxes indicate downregulated gene expression.

Notable similarities and differences in gene expression between the melanoma sEV types were noted for specific genes (Figure 6). A few genes were upregulated in two melanoma sEV types. Chemokine ligand (CXCL) 1, CXCL2, and CXCL8 were upregulated in both A-375 and SKMEL-28 sEVs. 

Other genes were downregulated in two or more melanoma sEV types. Decreased expression of B-cell lymphoma 2 like-1 (BCL2L1) and guanylate binding protein 1 (GBP1) was observed for SKMEL-28 and C-32 sEVs. Human leukocyte antigen (HLA)-A was downregulated in A-375 and SKMEL-28. Downregulation of HLA-C gene expression was the only finding common to all melanoma sEV types (A-375, SKMEL-28, and C-32) evaluated.

A few genes were either upregulated or downregulated depending on the sEV type. CXCL2 mRNA was increased in A-375 and SKMEL-28 sEVs but decreased in C-32 sEVs. Signal transducer and activator of transcription (STAT)-1 was upregulated in SKMEL-28 sEVs and downregulated in C-32 sEVs.

Detection of some mRNAs was unique to only one sEV type. HIF-1α, IL-1α, NFKB1, STAT3, and TP53 were only found to be significantly upregulated in A-375 sEVs. Upregulation of macrophage migration inhibitor factor (MIF), secreted phosphoprotein 1 (SPP1), and STAT1 were only observed for SKMEL-28 sEVs. Upregulation of Prostaglandin-Endoperoxide Synthase 2 (PTGS2, COX-2) was only noted for C-32 sEVs. 

**Figure 7:**
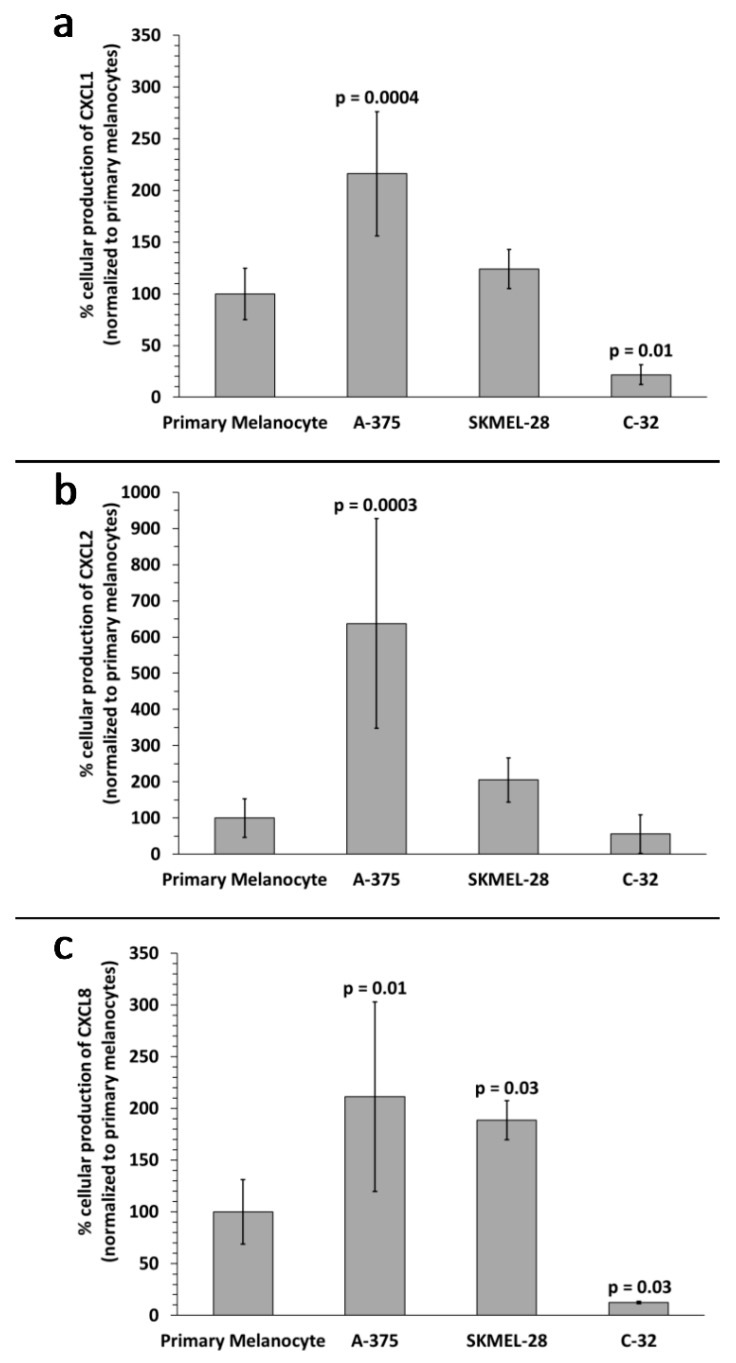
Melanoma cell versus primary melanocyte production of chemokine proteins. Percent protein production levels for (**a**) CXCL1, (**b**) CXCL2, and (**c**) CXCL8 by A-375, SKMEL-28, and C-32 melanoma cells compared to primary melanocytes (control, normalized to 100%) are shown. Data bars represent the average of (*n =* 4) independent experiments, error bars = SD, *p* values < 0.05 were considered statistically significant.

Upregulation of CXCL1, CXCL2, and CXCL8 mRNA in A-375 and SKMEL-28 melanoma sEVs was the most consistent finding (Figure 6). To determine whether CXCL1, CXCL2, and CXCL8 proteins were also produced by the melanoma cells, enzyme-linked immunosorbent assays (ELISAs) were performed on melanoma cell culture supernatants. The results demonstrate significantly increased production of CXCL1, CXCL2, and CXCL8 protein by A-375 melanoma cells compared to primary melanocytes (Figure 7). A trend toward increased production of CXCL1 and CXCL2 was also observed for SKMEL-28 cells versus primary melanocytes (Figure 7a,b). CXCL8 was significantly produced by SKMEL-28 versus primary melanocyte cells (Figure 7c). Increased production of CXCL8 protein by A-375 and SKMEL-28 melanoma cells was the most consistent finding (Figure 7c). Amelanotic C-32 melanoma cells produced less CXCL1, CXCL2, and CXCL8 than primary melanocytes (Figure 7).

## 3. Discussion

Biophysical characterization of the different sEV types demonstrated trending size and zeta potential differences between primary melanocyte sEVs and melanoma sEVs. In general, primary melanocyte sEVs were smaller and possessed lower negative, closer to neutral, zeta potentials. Such differences support the feasibility of assessing means to distinguish normal sEVs from melanoma sEVs or other tumor sEV types based on innate sEV biophysical properties [19,20]. 

To the best of our knowledge, this is the first study to investigate the presence of inflammation-related mRNA content in melanoma sEVs. Primary melanocyte sEVs proved to be an excellent reference for melanoma sEV inflammatory crosstalk mRNA content. However, it should be noted that the time required to cultivate primary melanocyte sEVs is not trivial. These primary cells grew extremely slow, as to be expected, and approximately six months was required to obtain between 1.4–2.0 mg of melanocyte sEV protein mass to conduct studies. In general, the melanoma sEV mRNA transcripts identified in this investigation can be classified as upregulated or downregulated in comparison to primary melanocyte sEV mRNA transcript content (Figure 6). 

Increased CXCL1, CXCL2, and CXCL8 mRNA in melanoma sEVs, relative to primary melanocyte sEVs, was the most consistent finding. These chemokines are pro-angiogenic and play important roles in the growth and metastasis of melanoma and other tumor types [21,22]. 

CXCL1, also known as melanoma growth stimulating activity alpha (MGSA-α), is an autocrine melanoma growth factor [23]. In contrast to primary melanocytes, MGSA-α can be constitutively overexpressed by melanoma cells in the absence of exogenous growth factors or serum [21]. 

CXCL1 and CXCL2 (MGSA-β, or macrophage inflammatory protein 2 alpha (MIP2- α)) are 90% identical in terms of their amino acid sequences [24]. Shi et al. reported that CXCL1 and CXCL2 produced by melanoma cells can facilitate melanoma survival by promoting expansion and recruitment of CD11b+ myeloid-derived suppressor cells (MDSCs) into tumors [24]. Within tumors, CD11b+ myeloid cells also served as an additional source of CXCL1 and CXCL2 production [24]. 

CXCL8, also known as interleukin 8 (IL-8), acts as a melanoma cell mitogen and growth factor [25]. IL-8 mediates the haptotactic migration of melanoma cells and production of matrix metalloprotease 2, further facilitating extracellular matrix degradation and melanoma migration [25]. CXCL8 is capable of inducing tumor stemness and maintaining, via an autocrine loop, the epithelial to mesenchymal (EMT) phenotype transition of tumor cells [26]. The mesenchymal phenotype facilitates tumor cell migration, invasion, and metastasis. 

Tumor-produced IL-8 also mediates cancer survival via paracrine immunosuppressive functions [27]. This includes recruitment of N2 polarized tumor-associated neutrophils (TANs) that in turn recruit pro-tumor regulatory T-cells (Tregs) and suppress adaptive anti-tumor T-cell immune function [27]. CXCL8 also recruits MDSCs that inhibit anti-tumor T cell function via the induction of Tregs and production of immunosuppressive cytokines including IL-10 and transforming growth factor beta (TGF-β) [27]. 

CXCL8/IL-8 expression is also produced by cells during senescence, or cell growth arrest [22]. Production of CXCL1 and CXCL8 by a variety of tumor cell types has been shown to be induced by the conditioned media obtained from senescent human peritoneal mesothelial cells [28,29]. Given the potential for conditioned media to harbor sEVs, coupled to the ability of sEVs to relay translatable mRNA to target cells [30], it is conceivable that inflammatory crosstalk mRNA found in melanoma sEVs may be of functional significance. Hypothetically, tumor sEV CXCL8 mRNA content might be adapted to serve as a useful biomarker for tumor senescence and monitoring potential for relapse during periods of remission. 

The only gene found to be overexpressed in C-32 melanoma sEVs compared to primary melanocytes was PTGS2/COX-2. A recent study determined that COX-2 expression positively correlates to PD-L1 expression by human melanoma cells [31]. The study concludes that the use of COX-2 inhibitors may augment the efficacy of anti-PD-L1 immunotherapy for melanoma. Conceivably, melanoma sEV COX-2 mRNA content might serve as a useful biomarker for predicting a given melanoma patient’s response to therapy. Further, we recently demonstrated that melanoma sEVs upregulate COX-2 in primary macrophages [9]. Induction of COX-2 in macrophages is a marker associated with the induction of pro-tumor, immunosuppressive M2b or M2d/tumor-associated macrophages [9]. Future investigations will explore whether melanoma sEV-associated COX-2 can be relayed to and expressed by macrophages. 

Downregulation of several mRNA transcripts in melanoma sEVs compared to primary melanocyte sEVs was observed as well. B-cell lymphoma 2 like 1 (BCL2L1/BCL-XL) and GBP1 were both downregulated in SKMEL-28 and C-32 sEVs. Increased expression of the anti-apoptotic protein BCL-XL is positively correlated to melanoma progression and phosphorylated STAT-3 (pSTAT-3) [32]. Interestingly, BCL-XL was not upregulated in A-375 melanoma sEVs. However, STAT-3 mRNA expression was increased in A-375 sEVs which is consistent with the relationship between BCL-XL and pSTAT-3. The STAT3 transcription factor induces anti-apoptotic, angiogenic, immunosuppressive, and metastatic gene expression profiles conducive to melanoma growth and survival [33]. In contrast, STAT1 activity antagonizes these same processes. Upregulation of STAT1 mRNA was detected only in SKMEL-28 sEVs. 

Downregulation of HLA-C mRNA in all three sEV types tested is particularly interesting given that melanoma cells are known to downregulate HLAs to prevent tumor immune surveillance [34,35,36]. It could be that the levels of HLA mRNAs present in circulating melanoma patient sEVs fluctuate over the course of the disease. A decrease may directly correlate to a lapsing tumor immune surveillance state. Monitoring HLA mRNA levels in sEVs would, therefore, translate into a minimally invasive means to monitor a given patient’s tumor immunogenicity. This would facilitate the selection of appropriate patient-specific immunotherapeutic treatment regimens. In future investigations, we plan to validate the diagnostic, prognostic, and/or predictive biomarker potential of the sEV shuttle mRNAs identified herein using melanoma patient-derived sEV samples. 

Comparing the inflammatory mRNA transcript profiles of the different melanoma sEV types might be used to categorize the melanoma sEV source cells in terms of their inflammation-associated pro-tumor attributes. For example, A-375 sEVs contained the most upregulated mRNA transcripts corresponding to the most known pro-tumor inflammatory functions. SKMEL-28 sEVs contained a mixture of pro-tumor and anti-tumor inflammatory mRNA transcripts. C-32 sEVs contained the least number of upregulated inflammatory mRNA transcripts and the highest number of downregulated mRNAs. Effectively, the inflammation-associated pro-tumor properties of melanoma sEVs may be relayed through their mRNA content. 

Because melanoma cells were once normal melanocytes, the strategy of using primary melanocyte sEVs as a control sEV population promises to streamline melanoma sEV biomarker discovery. This approach could potentially circumvent false positive leads associated with using less similar normal human plasma or serum-derived sEV reference populations. For example, inflammation-related mRNA candidates found in melanoma sEVs may be of functional or non-functional significance. Categorizing those of functional or non-functional significance may be possible if the normal inflammation-related mRNA content of primary melanocyte sEVs is known. 

With regard to biomarker development, excellent reviews by Liu et al. [37] and Vogel et al. [38] discuss how mRNA expression levels cannot be used to predict the degree of corresponding protein expression. In this study, we were able to correlate the expression of the most consistently upregulated A-375 and SKMEL-28 melanoma sEV mRNAs (CXCL1, CXCL2, and CXCL8) to CXCL1, CXCL2, and particularly CXCL8 protein production at the sEV source cell level. However, the degree of the observed upregulation of these melanoma sEV mRNAs was not identical to that of sEV source cell production of these chemokines. This finding might be explained in part by selective sorting of specific cellular RNAs into sEVs [39]. Additionally, or alternatively, this result may derive from multiple levels of transcriptional and translational regulation working in concert to produce each of the chemokines. 

It could be that assessing sEV chemokine mRNA content is a more consistent and reliable method to determine cellular chemokine production as opposed to evaluating the levels of chemokine protein, or free chemokine mRNA present in biofluids. The half-life of CXCL8 protein, for example, and other chemokines is brief at approximately 4 h [40]. The short half-life of cytokines and chemokines complicates their efficient and reliable detection and assessment in biological samples. This makes it difficult to predict the degree of chemokine protein expression based on chemokine RNA levels. Further, free RNA in biological fluids is rapidly degraded by ubiquitous plasma or serum RNAses [41]. The free RNA degradation process takes as little as 15 s [41]. In contrast, sEV membranes protect sEV mRNA from degradation [42] and provide for stable tracking of chemokine gene transcription. 

Ultimately, the presence of the identified inflammation-related mRNA transcripts in the melanoma sEVs is an intriguing basic science finding. It provides further insights into melanoma pathogenesis meriting future investigations. Small EV shuttle mRNA can be translated into target cells [43]. Conceivably, melanoma sEV chemokine mRNA might be of functional significance. It may be delivered to and translated in distal tissue microenvironments, facilitating lymphatic and extralymphatic pre-metastatic niche development and haptotactic tumor cell recruitment. In the future, pending the success of rigorous translational and clinical pilot studies, assessment of melanoma sEV inflammation-related mRNA transcript profiles might be a useful strategy to categorize the inflammatory state of melanoma patients. This will facilitate patient selection for immunotherapeutic trials, enable monitoring of responses to new biologic therapies, and increase the rate of discovery of new diagnostic, prognostic, and/or predictive melanoma biomarkers. 

## 4. Materials and Methods

### 4.1. Cell Culture 

Human melanoma cell lines HTB-72 (SKMEL-28) and CRL-1585 (C-32) were purchased from the American Type Culture Collection (ATCC, Manassas, VA, USA) and cryogenically preserved until use at passage numbers P20 or less for SKMEL-28, and P18 or less for C-32. The human melanoma cell line CRL-1619 (A-375) was obtained from the ATCC and was a gift provided by the Yaddanapudi laboratory. A-375 cells were cryogenically preserved until use at P16 or less. Human primary epidermal melanocytes (PCS-200-013) were purchased from the ATCC and cryogenically preserved until use at P6. Melanoma cells were cultured at 37 °C in 90% Dulbecco’s modified Eagle’s medium (DMEM) with 10% heat-inactivated fetal bovine serum (FBS) and 5% CO_2_. Primary melanocytes were cultured in primary melanocyte media composed of dermal basal medium (ATCC PCS-200-030), containing melanocyte growth factors (ATCC PCS-200-042), at 37 °C and 5% CO_2_.

### 4.2. Small EV Isolation by Standard Differential Centrifugation

Isolation of sEVs from cell culture by means of differential centrifugation has previously been established [5,18,44,45,46,47]. Briefly, to isolate melanoma sEVs, cells were grown to 70% confluence in three 300 cm^2^ flasks. Culture media was removed, and the cells were washed with DMEM. Melanoma cells were then cultured for 48 h in bovine sEV-free conditioned media. Conditioned media was prepared by subjecting normal culture media to overnight ultracentrifugation at 110,000× *g* to remove bovine sEVs [44]. Alternatively, given their slow growth characteristics, primary melanocytes were cultured continuously in bovine sEV-free conditioned primary melanocyte media. 

Post 48 h cellular growth in conditioned media, conditioned media was removed, diluted 1:1 in 50 mM trehalose (cryoprotectant) PBS [16], and processed using differential centrifugation with a Type 70 Ti rotor (Beckman Coulter Inc., Brea, CA, USA) [44]. Supernatants were collected after each round of centrifugation, and the pellets were discarded. Centrifugation at 3400× *g* for 30 min was used to remove residual cells, debris [5,48], and larger-sized EVs [4]. Subsequently, ultracentrifugation at 10,000× *g* for 30 min was used to remove medium-sized EVs [4]. Finally, the sEV pellet was collected after ultracentrifugation at 110,000× *g* for 1.5 h [9]. Small EVs were stored in 50 mM trehalose PBS cryoprotection media at −80 °C until further use [16].

### 4.3. Determination of sEV Size and Zeta Potential Properties

Small EVs were sized in 1× PBS using dynamic light scattering set to the lognormal number mode, and zeta potentials determined using a NanoBrook 90Plus (Brookhaven Instruments, Holtsville, NY, USA) nanoparticle tracking analyzer according to established methods [7,15,16,18,19]. Small EV protein concentrations were measured using a Pierce BCA protein assay (Thermo Fisher Scientific Inc., Waltham, MA, USA) as described previously [9,18,19,20,49].

### 4.4. Qualitative Determination of Melanoma sEV CD63 Marker Expression

Qualitative sEV CD63 marker expression was determined using an ExoELISA-ULTRA CD63 Kit (EXEL-ULTRA-CD63-1, System Biosciences, Palo Alto, CA, USA) according to the manufacturer’s instructions [9]. The kit enabled determination of whether primary melanocyte sEVs and melanoma sEVs qualitatively (positively versus negatively) expressed CD63. The kit used CD63 reference material to determine the level of CD63+ sEVs per μg of sEV protein using 10 μg of sEV protein starting material. Small EV CD63 expression was converted to percentiles (normalized to primary melanocyte CD63 expression, set to 100%) for purposes of comparison. 

### 4.5. Determination of sEV Density

For determination of sEV density on sucrose gradients, sEVs were labeled with the fluorescent red carbocyanine DiI (Invitrogen, Carlsbad, CA, USA) at a concentration of 1.0 mmol/L in 50 mM trehalose PBS and re-isolated at 110,000× *g* for 1.5 h according to established methods [16]. Flotation of DiI labeled sEVs on a continuous sucrose gradient (2.0–0.25 M sucrose, 20 mM HEPES/NaOH, pH 7.4) was performed similarly as previously described using a Beckman Coulter SW 41 rotor [16,18]. For each gradient, 400 μg of sEV protein was loaded except for the primary melanocyte sEVs in which case 80 μg of protein was loaded. The primary melanocytes grow very slowly, requiring multiple months of culture to accumulate small amounts of sEV material. The gradient was produced using a Gradient Master (Biocomp Instruments, Fredericton, NB, Canada) and was spun, after loading sEVs, for >15 h at 100,000× *g*. Post centrifugation, 1 mL fractions were collected from the bottom up. The density of each fraction was calculated using a refractometer [18]. Two hundred microliters of each fraction were added to a black 96-well plate, and DiI sEV fluorescence detected using a Tecan M200 infinite pro microplate reader according to established methods [16,18]. 

### 4.6. Detection of sEV Inflammation-Related mRNA Content by Quantitative Reverse Transcription PCR (RT-qPCR) 

Small EV RNA was isolated using Qiagen’s miRNAeasy kit (cat# 217004) according to the manufacturer’s instructions for purification of total RNA from cells according to established methods [9]. The protein mass of melanoma sEVs and melanocyte sEVs used for RNA isolation was ~1.4–2 mg. This required maintaining many of independent A375 and SKMEL-28 melanoma cell cultures for approximately 3 months to produce pooled sEV batches. Given their slower growth, sEV production required about 4 months for C-32 melanoma cells. Generating sEVs from primary melanocytes required the longest duration of cell culture, upwards of 6 months given their extremely slow growth properties. The Qiagen miRNAeasy kit was used rather than the more general Qiagen RNeasy kit because it allows for isolation of total RNA including mRNA and miRNA with one kit enabling future downstream miRNA analysis and saving precious sample materials. RNA quantity and quality were assessed using a Tecan M200 infinite pro microplate reader. For each sEV batch, 100 ng of total sEV RNA was converted to cDNA using Qiagen’s RT2 First Strand kit (cat#33041). Following conversion to cDNA, each sample was applied to a Qiagen Human Cancer Inflammation and Immunity Crosstalk qPCR Profiler Array (PAHS-181Z). RT-qPCR was performed using a StepOnePlus^TM^ Real-Time PCR system (Applied Biosystems Inc., Foster City, CA, USA). The beta-actin gene was identified as the best normalization gene across arrays. 

### 4.7. Enzyme-Linked Immunosorbent Assays (ELISAs)

The production of chemokine ligands CXCL1, CXCL2, and CXLC8 by melanoma cells and primary melanocytes was determined by ELISA of cell culture supernatants corresponding to the same cell culture and sEV isolation conditions described above (Methods Section 4.1, Section 4.2). Abcam human ELISA kits ab190805, ab184862, and ab214030 were used according to manufacturer’s instructions to detect cellular production of CXCL1, CXCL2, and CXCL8, respectively. 

### 4.8. Statistics

For sEV biophysical data sets, JMP version 14 (SAS Institute Inc., Cary, NC, USA) statistical software was utilized according to manufacturer instructions to perform one-way ANOVA between sEV data sets followed by Tukey’s Honest Significant Difference (HSD) test. Tukey’s HSD test was used to compare all possible pairs of means of sEV biophysical data sets to determine which pairs were statistically different assuming a normal sample distribution and an alpha of 0.05. For RT-qPCR Array results, significant differences in gene expression were determined using Qiagen’s online PCR array data analysis portal: The GeneGlobe Data Analysis Center [50]. For ELISA results, JMP version 14 (SAS Institute) statistical software was utilized to perform comparisons between each data pair using the Student’s t-test. 

## 5. Conclusions

Melanoma-derived sEVs contain inflammation-related mRNAs. This includes upregulation of chemokine ligand CXCL1, CXCL2, and CXCL8 mRNAs. CXCL1, CXCL2, and CXCL8 have known tumor-supportive functions. Inflammation-related mRNAs were identified using primary melanocyte-derived sEVs as a reference sEV population. A similar approach, comparing normal primary cell-derived sEV content to corresponding pathologic sEV content, might be applicable to the development of sEV-based biomarkers for other tumor types or diseases as well.
